# Non-Stationary Characteristics of AQM Based on the Queue Length

**DOI:** 10.3390/s23010485

**Published:** 2023-01-02

**Authors:** Andrzej Chydzinski

**Affiliations:** Department of Computer Networks and Systems, Silesian University of Technology, Akademicka 16, 44-100 Gliwice, Poland; andrzej.chydzinski@polsl.pl

**Keywords:** TCP/IP networks, active queue management, non-stationary characteristics, waiting time, number of losses

## Abstract

We performed a non-stationary analysis of a class of buffer management schemes for TCP/IP networks, in which the arriving packets were rejected randomly, with probability depending on the queue length. In particular, we derived formulas for the packet waiting time (queuing delay) and the intensity of packet losses as functions of time. These results allow us to observe how the evolution of the waiting time and losses depend on initial conditions (e.g., the full buffer) and system parameters (e.g., dropping probabilities, load, packet size distribution). As side results, the stationary waiting time and packet loss probability were obtained. Numerical examples demonstrate applicability of the theoretical results.

## 1. Introduction

Contemporary TCP/IP networks suffer from bufferbloat [1,2,3], i.e., frequent occurrences of long queues of packets in the output buffers of routers. Bufferbloat not only causes performance degradation of TCP/IP networks in terms of extending the mean packet delivery time and its variance, but has also unpleasant side effects. These include synchronization of high and low-intensity cycles among TCP connections and lockouts of TCP connections with long round-trip times [4].

To mitigate bufferbloat, various active queue management (AQM) algorithms for admission of packets arriving at the router’s buffer were proposed (see, e.g., [5,6,7,8,9] and the references therein). The central idea of these algorithms is that some arriving packets are rejected randomly, with a probability that evolves in time with evolving network conditions. This probability is, therefore, recomputed frequently, depending on the current or past buffer occupancies, packet rejection history, buffer overflow events, arrival rates, or other factors. Deleting some packets upon arrival not only prevents the growth of the queue length immediately, but also informs TCP sources about worsening network conditions and the need to reduce their sending rates. On the other hand, the random nature of the acceptance–rejection process reduces the synchronization of TCP connections.

AQM algorithms are also studied in the context of wireless sensor networks (WSNs) (see, e.g., [10,11,12,13,14] and the references therein). In WSNs, AQM is an effective means of congestion avoidance and mitigation of packet losses caused by buffer overflows. In fact, in some WSNs (e.g., in healthcare), buffer overflows and losses should be avoided even more than in wired TCP/IP networks (see the discussion in [11]).

An important group of AQM algorithms uses the current queue length to compute the rejection probability. Namely, the probability of rejecting a packet is a function of the length of the queue upon this packet’s arrival. For obvious reasons, this function should be non-decreasing. Several functions were proposed and tested in simulations so far, including a linear function, broken-linear, quadratic, a hybrid of cubic and linear, product of a linear and logarithmic one, and a beta function, see [15,16,17,18,19,20], respectively. Recently, such algorithms have been implemented in a prototype device and tested in a real network of a large university [21]. Besides wired TCP/IP networks, algorithms of this type are postulated for wireless sensor networks (see, e.g., [13,14], where a linear probability of dropping a packet is used).

In this paper, we studied the mathematical model of the AQM scheme with rejections based on the queue length. Such a model has already been studied in a few papers (the details will be given in the next section). The majority of these papers, however, are devoted to the stationary analysis of the queue length. Herein, we perform the non-stationary (transient) analysis of the model and study different, equally important characteristics, namely the waiting time and the number of losses. These characteristics are studied at the particular time *t*, which can be small or large. The main contributions of this paper are as follows:

- A new formula on the average virtual waiting time (workload) at the time *t* (Theorem 3);
- New formulas on the average number of packets lost in the interval (0,t) and the local intensity of losses at the time *t* (Theorems 5 and 6);
- New formulas on the stationary workload and loss probability (Theorems 4 and 7);
- New formulas on the joint distribution of the number of accepted packets and losses in a given interval (Theorems 1 and 2);
- Numerical examples computed for different initial states of the system, dropping probabilities, loads, and service time distributions.

Non-stationary characteristics provide deeper insight into the operation of a system, than their stationary counterparts, for several reasons. First of all, they are more general. From a non-stationary characteristic, it is usually easy to obtain the stationary one by letting t→∞, but not vice versa. Herein, we will obtain the stationary waiting time and loss probability as simple corollaries from general, non-stationary results. However, non-stationary results enable much more than that. For instance, they allow us to observe how long the system needs to converge to the steady state, how the convergence time depends on the system parameters, and the characteristics of interest depend on the initial conditions. All of these will be demonstrated herein in numerical examples. We will see how the system load, service time (packet size) distribution, and dropping probabilities influence the convergence time. Moreover, we will check the dependency of the state of the system shortly after the beginning of its operation, on the initial buffer occupancy.

It should be stressed that we are interested in the practical applicability of the theoretical results. Therefore, all the necessary auxiliary results are presented and discussed, making the theorems fully applicable in numerical calculations. In particular, we prove a theorem on the joint distribution of the number of accepted packets and losses in a given interval, when the service is suspended. This is not only needed in order to use the main theorems in practice, but is also an important characteristic on its own. We also recommend a method of inverting the Laplace transform. Finally, to make the results as widely applicable as possible, the service time distribution, which here is proportional to the packet size distribution, is general and may assume an arbitrary form. The function assigning dropping probabilities to queue lengths is also general. Therefore each of the aforementioned functions can be used, as well as any other.

The remainder of the article is structured in the following manner. In Section 2, we characterize previous work on queues with rejections based on the queue length. Then, the  model of interest is formally described in Section 3. In Section 4, the analytical framework used in the paper is sketched and an important auxiliary result is obtained, i.e., the joint distribution of the number of accepted and lost packets in a given interval, when the service is suspended. In Section 5, the transient analysis of the waiting time is carried out. The main result of this section is Theorem 3, on the Laplace transform of the transient virtual waiting time. As a side result, the stationary virtual waiting time is obtained in Theorem 4. In Section 6, the transient analysis of the number of lost packets is performed. The main results are Theorem 5, on the Laplace transform of the number of lost packets in the interval (0,t), and Theorem 6, on the intensity of losses at the time *t*. As a side result, the stationary loss probability is obtained in Theorem 7. In Section 7, all the steps needed to calculate the transient and stationary characteristics are systemized in an algorithmic manner. Then, in Section 8, numerical examples are presented and discussed. They demonstrate the transient behavior of the waiting time and losses, depending on dropping probabilities, queue length, system load and service time distribution. Furthermore, the theoretical results are verified by means of a discrete-event simulator. In Section 9, final conclusions are presented.

## 2. Related Work

To the best of the author’s knowledge, the results presented herein are new.

So far, majority of papers on the model of interest have been devoted to the stationary analysis of the queue length, under different assumptions on the arrival and service times. The model with exponential inter-arrival times and exponential service times was studied in [22,23,24,25]. In particular, the case with linear dropping probabilities was studied in [22], while the case with arbitrary dropping probabilities in [23,24]. In [25], a generalization, such that an arriving packet is accepted with probability depending on the available capacity of a continuous buffer, was considered. The model with general inter-arrival times and exponential service was studied in [26,27]. Namely, an approximate solution was presented in [26], while the exact solution in [27]. In [28], the system with general service times and exponential arrivals was studied, while in [29,30], its generalization to the continuous buffer. The transient queue length was investigated in [31,32,33], for a few types of the arrival process. Namely, a Poisson process was assumed in [31], a modulated Poisson process in [32] and a general renewal process in [33]. Finally, probability that in a given time interval the queue length is maintained under some levels was derived in [34].

Herein, we study different, equally important characteristics, i.e., the transient waiting time and intensity of losses. Note that in the non-stationary case, there is no simple relation between the average queue length and the waiting time. Little’s law, which constitutes a relation between the two, is valid only in the stationary case. Therefore, in the transient case separate formulas are required for the average queue length, waiting time, and loss intensity.

In this paper, a simplified model of traffic is used, i.e., the Poisson process. The reason why this process is used is that it makes the exact, transient analysis of the whole AQM model possible. The AQM model analyzed herein has several aforementioned features, which are of practical importance and have never been taken into account simultaneously. This comes with the cost of increased analytical complexity. In the literature, we can find analytical AQM models with much more detailed TCP traffic than herein, e.g., [35,36,37,38]. However, these models do not take into account some other properties of the system, which are taken into account herein. Moreover, they are approximate in nature—they treat network traffic as a continuous, rather than discrete, flow. There are also many TCP/AQM studies with very precise, discrete traffic models, but conducted via simulations only (e.g., [15,16,17,18,19,20]). Herein, we perform an exact, mathematical analysis of the discrete model, using tools and methods of the queuing theory.

## 3. Queuing Model

We are interested in a queuing model with one server (here an output link). Packets arrive according to the Poisson process of rate λ and form a queue if the server is busy upon arrival. The service time has an arbitrary distribution and its distribution function is denoted by F(t). The order of service of packets in the queue is not specified, it can be of any type.

Each arriving packet may not be permitted to enter the system. This happens randomly, with probability d(k), where *k* is the queue length on the arrival time of this packet. A rejected packet is deleted, so it is called a loss. We assume the finite buffer capacity with *K* positions in total, taking into account the service position. Therefore, function d(k) must fulfill the following assumption: d(k)=1 for k≥K. Besides the latter, function d(k) may have an arbitrary form.

The following notation will be of use. By X(t) we will denote the queue length at the time *t*. We adopt the convention that the length of the queue includes the service position, when this position is occupied. By $\bar{F}$, we denote the mean service time:

(1) $$\bar{F}=\int_{0}^{\infty }tdF\left(t\right),$$

and assume that $\bar{F}<\infty$. By  *S*, we denote the standard deviation of *F*. The load of the queue is defined as:

(2) $$\rho =\lambda \bar{F}.$$

Without loss of generality, we may assume that the time origin corresponds to the service completion epoch. Therefore, a new service is initiated at t=0, if only X(0)>0.

## 4. Analytical Framework and Auxiliary Results

The analytical framework used herein is based on tools and methods of the queuing theory and can be sketched as follows.

To find transient characteristics, we first build integral equations of the Volterra type. Then these equations are transformed into linear equations using the Laplace transform. The solutions of these linear equations are presented in a traditional way, using inverted square matrices. Finally, to move from the transform domain to the time domain, we use an inversion method for the Laplace transform.

To find stationary characteristics, we alter the last step, i.e., we do not invert the Laplace transform. Instead, we use the well-known property of the Laplace transform. Namely, if g(s) is the Laplace transform of function G(t), which has a limit as t→∞, then it holds:

$$\lim_{t\to \infty }G\left(t\right)=\lim_{s\to 0+}sg\left(s\right).$$

In practice, to obtain a stationary characteristic, we have to compute the value of sg(s) for a small, positive *s*.

Finally, when building integral equations, we need a special characteristic of the system, i.e., the joint distribution of the number of accepted packets and losses in a given interval, when the service is suspended. This characteristic will be found in this section.

Let $R_{n,i,j}\left(t\right)$ denote the probability that in the interval (0,t) the number of accepted packets is *i* and the number of lost packets is *j*, under the condition that no service is completed in (0,t) and X(0)=n. Let $Q_{n,i}\left(t\right)$ denote the probability that in the interval (0,t) the number of accepted packets is *i*, given that no service is completed in (0,t) and X(0)=n.

The problem of finding $Q_{n,i}\left(t\right)$ was already solved in article [28]. Namely, it was shown that:

(3) $$q_{n,i}\left(s\right)=\frac{\prod_{j=0}^{i-1}\left[\lambda (1-d(n+j))\right]}{\prod_{j=0}^{i}\left[s+\lambda (1-d(n+j))\right]},$$

where

(4) $$q_{n,i}\left(s\right)=\int_{0}^{\infty }e^{-st}Q_{n,i}\left(t\right)dt.$$

Formula (3) allows us to find effectively $Q_{n,i}\left(t\right)$ using one of the methods of the Laplace transform inversion.

Characteristic $R_{n,i,j}\left(t\right)$ has not been derived so far. Therefore, we start with proving the following theorem.

**Theorem 1.**  *For every n≥0, it holds:*

(5) $$r_{n,0,0}\left(s\right)=\frac{1}{s+\lambda },$$

(6) $$r_{n,i,0}\left(s\right)=\left(1-d\left(n\right)\right)r_{n+1,i-1,0}\left(s\right)\frac{\lambda }{s+\lambda },\;\;\;\;i\geq 1,$$

(7) $$r_{n,0,j}\left(s\right)=d\left(n\right)r_{n,0,j-1}\left(s\right)\frac{\lambda }{s+\lambda },\;\;\;\;j\geq 1,$$

(8) $$r_{n,i,j}\left(s\right)=\left(1-d\left(n\right)\right)r_{n+1,i-1,j}\left(s\right)\frac{\lambda }{s+\lambda }+d\left(n\right)r_{n,i,j-1}\left(s\right)\frac{\lambda }{s+\lambda },\;\;\;\;i\geq 1,j\geq 1,$$

*where*

(9) $$r_{n,i,j}\left(s\right)=\int_{0}^{\infty }e^{-st}R_{n,i,j}\left(t\right)dt.$$

**Proof.**  Assume an arbitrary n≥0. First, note that in order to have no accepted packets and no losses by the time *t*, there must be no arrivals by the time *t*. Therefore, the first arrival must happen after *t*. Hence:

(10) $$R_{n,0,0}\left(t\right)=e^{-\lambda t}.$$

Second, to have at least one accepted packet and no losses by the time *t*, there must be a packet arrival by the time *t* and this packet must be accepted. Thus, conditioning on the first arrival time, *u*, we have:

(11) $$R_{n,i,0}\left(t\right)=\left(1-d\left(n\right)\right)\int_{0}^{t}\lambda e^{-\lambda u}R_{n+1,i-1,0}\left(u\right)du,\;\;\;i\geq 1.$$

Similarly, to have no accepted packets and at least one loss by the time *t*, there must be a packet arrival by the time *t* and this packet must be lost. Therefore, conditioning on the first arrival time, *u*, yields:

(12) $$R_{n,0,j}\left(t\right)=d\left(n\right)\int_{0}^{t}\lambda e^{-\lambda u}R_{n,0,j-1}\left(u\right)du,\;\;\;j\geq 1.$$

Finally, to have at least one accepted packet or at least one loss by the time *t*, there must be a packet arrival by the time *t*, which can be accepted or lost. In this case we obtain:

(13) $$R_{n,i,j}\left(t\right)=\left(1-d\left(n\right)\right)\int_{0}^{t}\lambda e^{-\lambda u}R_{n+1,i-1,j}\left(u\right)du+d\left(n\right)\int_{0}^{t}\lambda e^{-\lambda u}R_{n,i,j-1}\left(u\right)du,\;\;\;\;i\geq 1,j\geq 1.$$

Now the proof can be completed easily. Applying the Laplace transform to (10)–(13) yields (5)–(8), respectively.    □

Recursive formulas (5)–(8) can be also combined in an explicit formula for $r_{n,i,j}\left(s\right)$ in the following theorem.

**Theorem 2.**  *For every n≥0, i≥0, j≥0 it holds:*

(14) $$r_{n,i,j}\left(s\right)=\left(\frac{\lambda^{i+j}}{{\left(s+\lambda \right)}^{i+j+1}}\prod_{k=0}^{i-1}\left(1-d\left(k+n\right)\right)\right)\left(\sum_{\begin{array}{c} m_{0},\ldots ,m_{i}\geq 0,\\ m_{0}+\ldots +m_{i}=j \end{array}}\prod_{l=0}^{i}d^{m_{l}}\left(n+l\right)\right).$$

The proof can be carried out using formulas (5)–(8) and mathematical induction.

Now, $R_{n,i,j}\left(t\right)$ can be found effectively using either the recursive formulas (5)–(8), or the explicit formula (14), combined with one of the methods of the Laplace transform inversion. In practice, (5)–(8) are more useful than (14). They enable fast calculation of $r_{n,i,j}\left(s\right)$ for arbitrary *s* and a quick inversion of the transform. Formula (14), although having a compact form, is computationally more demanding than (5)–(8), due to the need of finding all divisions $m_{0},\ldots ,m_{i}\geq 0$, such that $m_{0}+\ldots +m_{i}=j$.

## 5. Waiting Time

Let $V_{n}\left(t\right)$ denote the average virtual waiting time at the time *t*, assuming X(0)=n. This is the average time a hypothetical packet entered the system at the time *t*, was accepted, and would spend in the queue before the service. In other words, $V_{n}\left(t\right)$ is the average unfinished work (workload) at the time *t*. Naturally, $V_{n}\left(t\right)$ is a non-stationary characteristic, which depends on time and the initial buffer occupancy, X(0).

The main purpose of this section is to derive the Laplace transform of $V_{n}\left(t\right)$, i.e.,

(15) $$v_{n}\left(s\right)=\int_{0}^{\infty }e^{-st}V_{n}\left(t\right)dt.$$

Assume first 1≤n≤K. In such case, a new service is initiated at the time t=0. Using the formula of total probability with respect to the completion time of this service, *u*, we can build the following integral equation:

(16) $$\begin{array}{cc} V_{n}\left(t\right)= & \sum_{k=0}^{K-n}\int_{0}^{t}Q_{n,k}\left(u\right)V_{n+k-1}\left(t-u\right)dF\left(u\right)\\ \; & +\sum_{k=0}^{K-n}Q_{n,k}\left(t\right)\int_{t}^{\infty }\left[u-t+\left(n+k-1\right)\bar{F}\right]dF\left(u\right). \end{array}$$

In particular, the first part of (16) covers the case where the first service is completed by the time *t*. In this case, the new queue length at the time *u* is n+k−1 with probability $Q_{n,k}\left(u\right)$, defined in the previous section. The second part covers the case where the first service is completed after *t*. In that case, there are n+k packets in the system at the time *t*, with probability $Q_{n,k}\left(t\right)$. Moreover, one of these packets is already in service, and the remaining service time is u−t. Therefore, to compute the average workload at *t*, we have to sum u−t and n+k−1 full service times, each on average equal to $\bar{F}$.

Assume now n=0. In such a case, the system waits for the first arrival to initiate the first service. Using the formula of total probability with respect to the first arrival time, *v*, we now have the following integral equation:

(17) $$V_{0}\left(t\right)=\left(1-d(0)\right)\int_{0}^{t}\lambda e^{-\lambda v}V_{1}\left(t-v\right)dv+d\left(0\right)\int_{0}^{t}\lambda e^{-\lambda v}V_{0}\left(t-v\right)dv.$$

The first part of (17) covers the case where the first packet arrives by the time *t* and is permitted to enter. Therefore, the new queue length at the time *v* is 1. The second part covers the case where the first packet arrives by the time *t* and is rejected. Therefore, the queue length remains 0 at the time *v*. In the case, where the first packet arrives after *t*, the queue at the time *t* is empty. Hence, the workload is zero and such a case does not have to be included in (17).

Integrating by parts the second integral of (16), we have:

(18) $$\int_{t}^{\infty }\left[u-t+\left(n+k-1\right)\bar{F}\right]dF\left(u\right)=\left(n+k-1\right)\bar{F}\left[1-F\left(t\right)\right]+g\left(t\right),$$

with

(19) $$g\left(t\right)=\int_{0}^{\infty }\left(1-F\left(v+t\right)\right)dv.$$

Thus, from (16) and (18), we have:

(20) $$\begin{array}{cc} V_{n}\left(t\right)= & \sum_{k=0}^{K-n}\int_{0}^{t}Q_{n,k}\left(u\right)V_{n+k-1}\left(t-u\right)dF\left(u\right)\\ \; & +\sum_{k=0}^{K-n}\left[\left(n+k-1\right)\bar{F}Q_{n,k}\left(t\right)\left(1-F\left(t\right)\right)+Q_{n,k}\left(t\right)g\left(t\right)\right],\;\;\;\;1\leq n\leq K. \end{array}$$

Applying the Laplace transform to (20), we obtain:

(21) $$v_{n}\left(s\right)=\sum_{k=0}^{K-n}b_{n,k}\left(s\right)v_{n+k-1}\left(s\right)+h_{n}\left(s\right),\;\;\;\;1\leq n\leq K,$$

where

(22) $$b_{i,j}\left(s\right)=\int_{0}^{\infty }e^{-st}Q_{i,j}\left(t\right)dF\left(t\right),$$

(23) $$c_{i,j}\left(s\right)=\int_{0}^{\infty }e^{-st}Q_{i,j}\left(t\right)\left(1-F\left(t\right)\right)dt,$$

(24) $$y_{i,j}\left(s\right)=\int_{0}^{\infty }e^{-st}Q_{i,j}\left(t\right)g\left(t\right)dt,$$

(25) $$h_{n}\left(s\right)=\sum_{k=0}^{K-n}\left[\left(n+k-1\right)\bar{F}c_{n,k}\left(s\right)+y_{n,k}\left(s\right)\right].$$

We may also apply the Laplace transform to (17), which yields:

(26) $$v_{0}\left(s\right)=v_{1}\left(s\right)\frac{(1-d(0))\lambda }{\lambda +s}+v_{0}\left(s\right)\frac{d(0)\lambda }{\lambda +s}.$$

Now we can rewrite systems (21), and (26) in matrix forms. Namely, introducing column vectors v(s) and h(s):

(27) $$v\left(s\right)={\left[v_{0}\left(s\right),\ldots ,v_{K}\left(s\right)\right]}^{T},$$

(28) $$h\left(s\right)={\left[0,-h_{1}\left(s\right),\ldots ,-h_{K}\left(s\right)\right]}^{T},$$

and matrix A(s):

(29) $$A\left(s\right)={\left[a_{ij}\left(s\right)\right]}_{i,j=0,\ldots ,K},\;\;\;a_{ij}\left(s\right)=\left\{\begin{array}{cc} d(0)\lambda /(\lambda +s), & \mathrm{if}\;\;i=j=0,\\ (1-d(0))\lambda /(\lambda +s), & \mathrm{if}\;\;i=0,j=1,\\ b_{i,j-i+1}\left(s\right), & \mathrm{if}\;\;1\leq i\leq K,i-1\leq j\leq K-1,\\ 0, & \mathrm{otherwise}, \end{array}\right.$$

from (21) and (26) we obtain:

(30) (A(s)−I)v(s)=h(s),

where *I* is the identity matrix. Therefore, we have proven the following theorem.

**Theorem 3.**  *The Laplace transform of the average virtual waiting time (workload) at the time t equals:*

(31) $$v\left(s\right)={\left(A\left(s\right)-I\right)}^{-1}h\left(s\right),$$

*where A(s) is given in (29), while h(s) in (28) and (25).*

From Theorem 3 we can obtain easily the average workload in the stationary regime, *V*. Namely, using properties of the Laplace transform we have:

(32) $$V=\lim_{t\to \infty }V_{0}\left(t\right)=\lim_{s\to 0+}s{\left[v\left(s\right)\right]}_{1},$$

where ${\left[\;\right]}_{1}$ denotes the first element of a vector. Obviously, any other element can be used, because the stationary workload is independent of the initial queue length, *n*. Combining (31) and (32) we obtain the following theorem.

**Theorem 4.**  *The stationary average virtual waiting time equals:*

(33) $$V=\lim_{s\to 0+}s{\left[{\left(A\left(s\right)-I\right)}^{-1}h\left(s\right)\right]}_{1}.$$

## 6. Number of Losses

Let $L_{n}\left(t\right)$ denote the average number of packets lost by the time *t*, assuming X(0)=n. Let $l_{n}\left(t\right)$ be its Laplace transform, i.e.,:

(34) $$l_{n}\left(s\right)=\int_{0}^{\infty }e^{-st}L_{n}\left(t\right)dt.$$

Firstly, let us assume 1≤n≤K. Using the law of total probability with respect to the completion time of the first service, *u*, we have:

(35) $$\begin{array}{cc} L_{n}\left(t\right)= & \sum_{k=0}^{K-n}\sum_{j=0}^{\infty }\int_{0}^{t}R_{n,k,j}\left(u\right)\left[j+L_{n+k-1}\left(t-u\right)\right]dF\left(u\right)\\ \; & +\sum_{k=0}^{K-n}\sum_{j=0}^{\infty }jR_{n,k,j}\left(t\right)\left[1-F\left(t\right)\right]. \end{array}$$

The first double sum in (35) covers the situation, where the first service is completed by the time *t*. In such a situation, there are *k* acceptances and *j* losses by the time *u* with probability $R_{n,k,j}\left(u\right)$, defined and computed in Section 4. The new queue length at the time *u* is n+k−1, thus the average number of losses at the time *t* must equal now to *j* (already lost packets) plus $L_{n+k-1}\left(t-u\right)$. The second double sum in (35) covers the situation, where the first service is completed after *t*, which happens with probability 1−F(t). In that situation, there are *k* acceptances and *j* losses with probability $R_{n,k,j}\left(t\right)$ by the time *t*.

Secondly, let us assume n=0. Using the law of total probability with respect to the first arrival time, *v*, w have:

(36) $$L_{0}\left(t\right)=\left(1-d\left(0\right)\right)\int_{0}^{t}\lambda e^{-\lambda v}L_{1}\left(t-v\right)dv+d\left(0\right)\int_{0}^{t}\lambda e^{-\lambda v}\left[1+L_{0}\left(t-v\right)\right]dv.$$

The first integral in (36) covers the situation, where the first packet arrives by the time *t* and is accepted, while the second integral involves the situation where the first packet arrives by the time *t* and is rejected. If there are no arrivals by the time *t*, then the number of packets lost by the time *t* is zero, and this situation can be omitted in (36).

Application of the Laplace transform to (35) yields:

(37) $$l_{n}\left(s\right)=\sum_{k=0}^{K-n}\sum_{j=0}^{\infty }w_{n,k,j}\left(s\right)\left[\frac{j}{s}+l_{n+k-1}\left(s\right)\right]+\sum_{k=0}^{K-n}\sum_{j=0}^{\infty }jx_{n,k,j}\left(s\right),\;\;\;\;1\leq n\leq K,$$

where

(38) $$w_{n,i,j}\left(s\right)=\int_{0}^{\infty }e^{-st}R_{n,i,j}\left(t\right)dF\left(t\right),$$

(39) $$x_{n,i,j}\left(s\right)=\int_{0}^{\infty }e^{-st}R_{n,i,j}\left(t\right)\left(1-F\left(t\right)\right)dt.$$

From the definitions of $R_{n,i,j}\left(t\right)$ and $Q_{n,i}\left(t\right)$ given in Section 4, it follows:

(40) $$\sum_{j=0}^{\infty }w_{n,k,j}\left(s\right)=b_{n,k}\left(s\right),$$

where $b_{n,k}\left(s\right)$ is defined in (22). Therefore, from (37) and (40), we obtain:

(41) $$l_{n}\left(s\right)=\sum_{k=0}^{K-n}b_{n,k,j}\left(s\right)l_{n+k-1}\left(s\right)+z_{n}\left(s\right),\;\;\;\;1\leq n\leq K,$$

with

(42) $$z_{n}\left(s\right)=\sum_{k=0}^{K-n}\sum_{j=0}^{\infty }j\left[\frac{w_{n,k,j}\left(s\right)}{s}+x_{n,k,j}\left(s\right)\right].$$

Application of the Laplace transform to (36) gives:

(43) $$l_{0}\left(s\right)=l_{1}\left(s\right)\frac{(1-d(0))\lambda }{\lambda +s}+l_{0}\left(s\right)\frac{d(0)\lambda }{\lambda +s}+\frac{d(0)\lambda }{\lambda s+s^{2}}.$$

The system of Equations (41) and (43) can be presented now in the matrix form. Namely, we have:

(44) (A(s)−I)l(s)=z(s),

where

(45) $$l\left(s\right)={\left[l_{0}\left(s\right),\ldots ,l_{K}\left(s\right)\right]}^{T},$$

(46) $$z\left(s\right)={\left[-\frac{d(0)\lambda }{\lambda s+s^{2}},-z_{1}\left(s\right),\ldots ,-z_{K}\left(s\right)\right]}^{T},$$

and matrix A(s) is defined in (29).

Finally, from (44), we obtain the following theorem.

**Theorem 5.**  *The Laplace transform of the average number of packets lost by the time t equals:*

(47) $$l\left(s\right)={\left(A\left(s\right)-I\right)}^{-1}z\left(s\right),$$

*where A(s) is given in (29), while z(s) in (46) and (42).*

The average number of losses by the time *t* is, obviously, a non-decreasing function of time. For a more intuitive description of the transient loss process, it is perhaps better to use the intensity of losses, $I_{n}\left(t\right)$, defined as:

(48) $$I_{n}\left(t\right)=\frac{dL_{n}\left(t\right)}{dt}.$$

Having Theorem 5, we can easily obtain the Laplace transform of the intensity of losses. Denote:

(49) $$i_{n}\left(s\right)=\int_{0}^{\infty }e^{-st}I_{n}\left(t\right)dt,\;\;\;i\left(s\right)={\left[i_{0}\left(s\right),\ldots ,i_{K}\left(s\right)\right]}^{T},$$

Using (47) and the basic properties of the Laplace transform, we arrive at the following theorem.

**Theorem 6.**  *The Laplace transform of the intensity of losses at the time t equals:*

(50) $$i\left(s\right)=s{\left(A\left(s\right)-I\right)}^{-1}z\left(s\right).$$

We can also obtain the stationary loss probability, i.e.,

(51) $$p_{L}=\lim_{t\to \infty }\frac{I_{0}\left(t\right)}{\lambda }.$$

Using again properties of the Laplace transform, we have:

(52) $$\lim_{t\to \infty }I_{0}\left(t\right)=\lim_{s\to 0+}s{\left[i\left(s\right)\right]}_{1}.$$

Finally, from (50)–(52), we have the following theorem.

**Theorem 7.**  *The stationary loss probability equals:*

(53) $$p_{L}=\frac{1}{\lambda }\lim_{s\to 0+}s^{2}{\left[{\left(A\left(s\right)-I\right)}^{-1}z\left(s\right)\right]}_{1}.$$

## 7. Calculation Procedures

In this section, we will systemize all the steps needed to perform calculations based on the proven theorems.

Before that, note that in order to obtain transient results in the time domain, we have to apply a method of numerical inversion of the Laplace transform. A very efficient method, in terms of the computation time, yet accurate enough for most practical purposes, is the Zakian method [39]. Namely, assuming that g(s) is the Laplace transform of function G(t), the Zakian formula states:

(54) Gt≈2t−1∑j=15Rewjg(uj/t),

where coefficients $w_{j}$ and $u_{j}$ are given in Table 1.

Now we can present the final computational procedures. Firstly, assume that we want to obtain the average waiting time, the average number of losses, and the average intensity of losses, at some particular time $t_{0}$, given that the initial queue length was $n_{0}$. The procedure is sketched in Algorithm 1.

Finally, we should extract the entry number $n_{0}$ of each resulting vector, V, L, and I, to obtain the average waiting time, the average number of losses, and the average intensity of losses at time $t_{0}$, respectively, for the initial queue length $n_{0}$.
**Algorithm 1** Transient characteristics1:**procedure**2:    $t:=t_{0}$3:    **for** j=1,⋯,5 **do**4:        $s_{j}:=u_{j}/t$, where $u_{j}$ is given in Table 15:        compute matrix $A_{j}:=A\left(s_{j}\right)$ using formula (29)6:        compute vector $h_{j}:=h\left(s_{j}\right)$ using formulas (28) and (25)7:        compute vector $z_{j}:=z\left(s_{j}\right)$ using formulas (46) and (42)8:        compute vector $v_{j}:={\left(A_{j}-I\right)}^{-1}h_{j}$9:        compute vector $l_{j}:={\left(A_{j}-I\right)}^{-1}z_{j}$10:       compute vector $i_{j}:=s_{j}l_{j}$11:    compute vector V:=2t−1∑j=15Rewjvj, where $w_{j}$ is given in Table 112:    compute vector L:=2t−1∑j=15Rewjlj13:    compute vector I:=2t−1∑j=15Rewjij14:    **close**;

Stationary characteristics are easier to obtain because the inversion of the Laplace transform is not required. Instead, we have to compute the limits in (33) and (53), by using a small value of *s*. In most practical applications, $s=10^{-8}$ provides sufficiently accurate results. The procedure for calculating the stationary average waiting time and stationary loss probability is sketched in the following Algorithm 2.
**Algorithm 2** Stationary characteristics1:**procedure**2:    $s:=10^{-8}$3:    compute matrix A:=A(s) using formula (29)4:    compute vector h:=h(s) using formulas (28) and (25)5:    compute vector z:=z(s) using formulas (46) and (42)6:    compute vector $V:=s{\left(A-I\right)}^{-1}h$7:    compute vector $p:=\frac{s^{2}}{\lambda }{\left(A-I\right)}^{-1}z$8:    **close**;

From the resulting vector V, we should extract the first entry to obtain the stationary average waiting time. To obtain the stationary loss probability, we take the first entry of vector p.

## 8.  Examples

In this section, we will present a few numerical examples demonstrating the evolution in time of the virtual waiting time and intensity of losses, depending on the aggressiveness of function d(k), the initial buffer occupancy, and queue load and service time distribution ($d_{1}\left(k\right)$ is more aggressive than $d_{2}\left(k\right)$ if $d_{1}\left(k\right)\geq d_{2}\left(k\right)$ for every *k*).

The service time distribution is hyperexponential, with the following parameters: $\left(p_{1},p_{2}\right)=\left(\frac{4}{5},\frac{1}{5}\right)$, and $\left(\mu_{1},\mu_{2}\right)=\left(2,\frac{1}{3}\right)$. It can be checked that the mean service time is 1, while the standard deviation is 1.73, which is a moderate value, but significantly higher than in the exponential distribution. This service time distribution is altered only in Section 8.4, where distributions with larger deviations are considered as well.

The arrival rate is 1.1, resulting in a slightly overloaded queue, ρ=1.1. This is altered only in Section 8.3, where strongly overloaded and strongly underloaded queues are considered as well.

### 8.1. Function d(k)

In this subsection, we study the effect of the following five functions d(k) on the evolution of the virtual waiting time and intensity of losses:

(55) $$\begin{array}{cc} d_{1}\left(k\right) & =\left\{\begin{array}{ccc} 0, & \mathrm{if} & k<15,\\ (k-15)/15, & \mathrm{if} & 15\leq k<30,\\ 1, & \mathrm{if} & k\geq 30, \end{array}\right. \end{array}$$

(56) $$\begin{array}{cc} d_{2}\left(k\right) & =d_{1}^{2}\left(k\right), \end{array}$$

(57) $$\begin{array}{cc} d_{3}\left(k\right) & =\sqrt{d_{1}\left(k\right)}, \end{array}$$

(58) $$\begin{array}{cc} d_{4}\left(k\right) & =\left\{\begin{array}{ccc} \frac{1}{2}d_{1}\left(k\right), & \mathrm{if} & k<30,\\ 1, & \mathrm{if} & k\geq 30, \end{array}\right. \end{array}$$

(59) $$\begin{array}{cc} d_{5}\left(k\right) & =d_{1}\left(k+5\right). \end{array}$$

**Figure 1:**
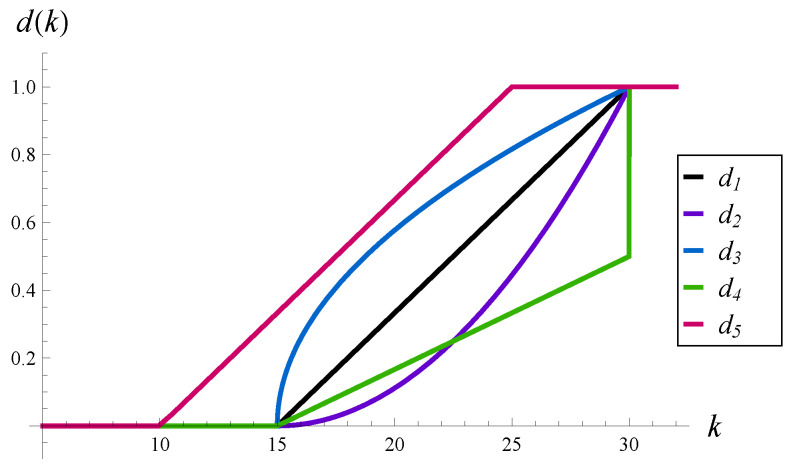
Functions $d_{1}$–$d_{5}$.

These functions are depicted in Figure 1. The following idea is behind such a choice. The linear function $d_{1}$ is the starting point. Functions $d_{2}$ and $d_{3}$ operate on the same interval as $d_{1}$, but $d_{2}$ is less aggressive than $d_{1}$ and convex, while $d_{3}$ is more aggressive than $d_{1}$ and concave. Therefore, comparing the results for $d_{1}$–$d_{3}$, we can study the impact of the aggressiveness of function *d* in terms of its convexity. The next function, $d_{4}$, operates on the same interval as $d_{1}$, but has dropping probabilities scaled by the factor of $\frac{1}{2}$. Thus comparing the results for $d_{1}$ and $d_{4}$, we can study the impact of aggressiveness of function *d*, understood as the scaling factor. Finally, $d_{5}$ has the same form as $d_{1}$, but operates on a different interval, i.e., it begins to reject packets more quickly than $d_{1}$. Therefore, comparing the results for $d_{1}$ and $d_{5}$, we can observe the impact of aggressiveness of function *d* in terms of its attack point.

**Figure 2:**
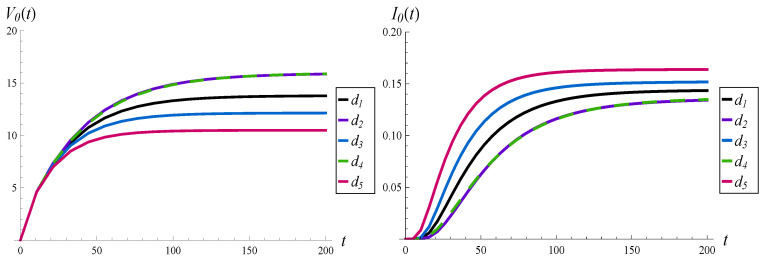
Average waiting time (on the left) and intensity of losses (on the right) vs. time, for different functions d(k).

In Figure 2, the transient virtual waiting time and the intensity of losses are depicted for functions $d_{1}$–$d_{5}$ and an initially empty buffer, X(0)=0.

It is easy to observe that the waiting times and intensity losses converge to stationary values in every case. For a particular function *d*, the convergence times are roughly the same for the waiting time and intensity of losses. However, convergence times differ among them. Namely, the system stabilizes around t=90 if $d_{5}$ is used, around t=120 if $d_{3}$ is used, around t=150 if $d_{1}$ is used, and around t=200 if $d_{2}$ or $d_{4}$ is used.

It is clear that the more aggressive function *d*, the shorter the convergence time. It is not crucial whether “more aggressive” means “more concave” or a “smaller attack point”.

Especially interesting are functions $d_{2}$ and $d_{4}$. The waiting times and loss intensities for $d_{2}$ and $d_{4}$ are practically identical, on the whole time axis. This effect is quite surprising, given that $d_{2}$ and $d_{4}$ differ significantly from each other (see Figure 1). It is already known that the same value of a characteristic can be obtained by applying substantially different functions *d*, but only in the stationary regime, for t=∞. It has not been demonstrated yet that this can be achieved also on the whole time axis, t∈(0,∞).

### 8.2. Initial Buffer Occupancy

In this subsection, we study the effect of the initial buffer occupancy, X(0)=n, on the evolution of the virtual waiting time and intensity of losses.

**Figure 3:**
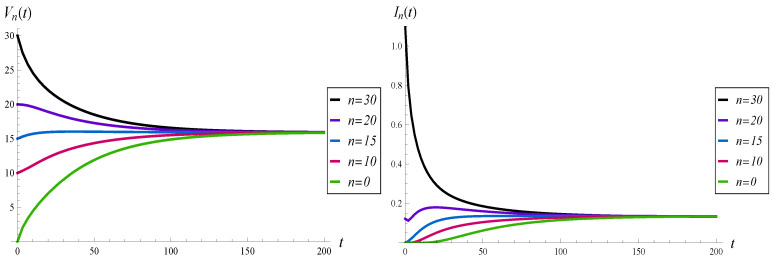
Average waiting time (on the left) and intensity of losses (on the right) vs. time, for different initial buffer occupancies.

**Figure 4:**
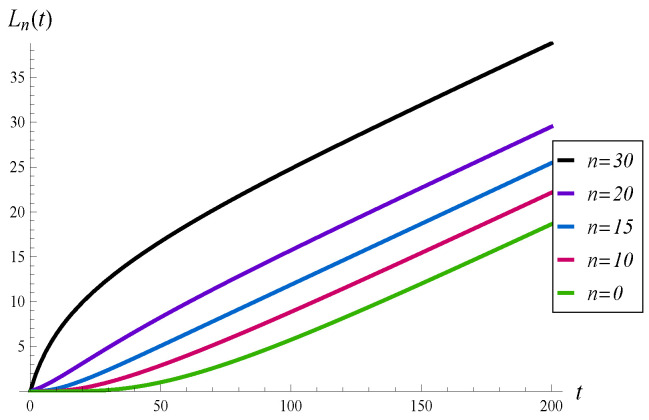
Average number of losses in (0,t) for different initial buffer occupancies.

The results for n=0, 10, 15, 20, and 30 are depicted in Figure 3 and Figure 4. In this comparison, function $d_{2}$ is used.

As we can see, all the curves in Figure 3 converge to the same, steady-state level, in a similar time. In the same way, all the curves in Figure 4 achieve the same gradient. For n=30, the initial intensity of losses equals the arrival rate, i.e., $I_{30}\left(0+\right)=\lambda =1.1$. This is due to the fact that $d_{2}\left(k\right)=1$ for k=30, which means that just after the start of the system, every arriving packet is lost.

**Figure 5:**
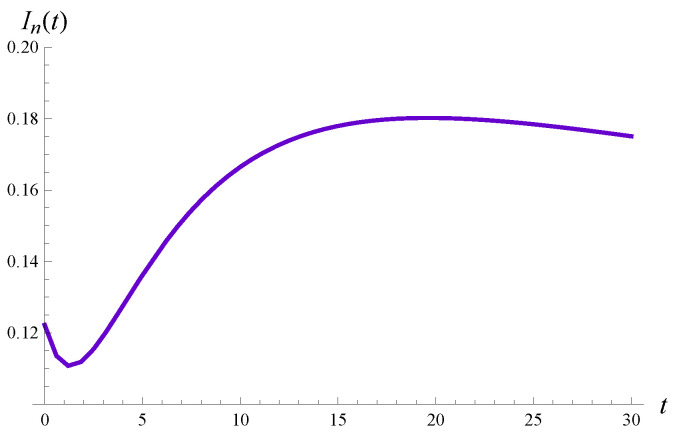
The intensity of losses vs. time for n=20.

It is interesting that for n=20 the intensity of losses has a non-monotonic form, with two extrema. This is shown in detail in Figure 5, in which the time interval (0,30) is enlarged.

### 8.3. Queue Load

In this subsection, we check the effect of the queue load, ρ, on the evolution of the virtual waiting time and intensity of losses. In particular, an underloaded queue with ρ=0.5, a critically loaded queue with ρ=1 and an overloaded queue with ρ=1.5, are tested. Moreover, X(0)=30 and function $d_{4}$ are assumed.

**Figure 6:**
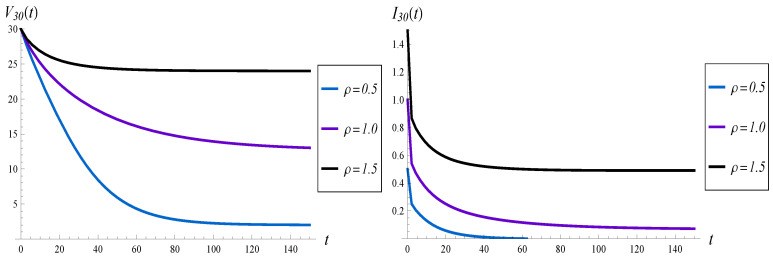
Average waiting time (on the left) and intensity of losses (on the right) vs. time, for different loads of the queue.

**Figure 7:**
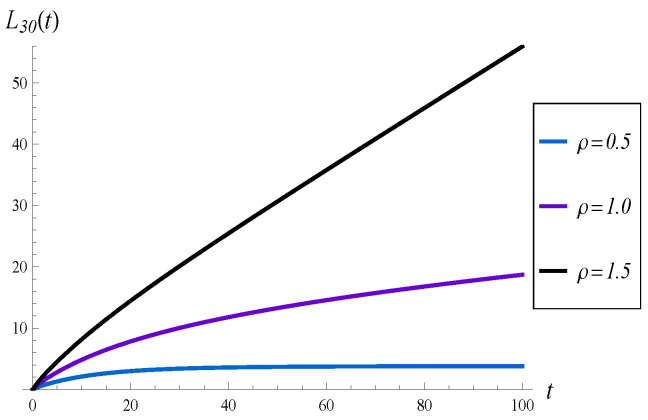
Average number of losses in (0,t) for different loads of the queue.

The results are shown in Figure 6 and Figure 7. The most interesting observation is that for ρ=1 the convergence time is the longest among the three. Namely, when ρ=0.5 or ρ=1.5, the system becomes stable at around t=100, while for ρ=1, it is not stable even at t=150. Therefore, the convergence time to the steady state is not a monotonic function of the load.

### 8.4. Service Time Distribution

In this final subsection, we test the effect of the service time distribution on the evolution of the virtual waiting time and intensity of losses. In particular, we use three hyperexponential service time distributions, with the following parameters:

$F_{1}$: $\left(p_{1},p_{2}\right)=\left(\frac{4}{5},\frac{1}{5}\right)$ and $\left(\mu_{1},\mu_{2}\right)=\left(2,\frac{1}{3}\right)$,
$F_{2}$: $\left(p_{1},p_{2}\right)=\left(\frac{24}{25},\frac{1}{25}\right)$ and $\left(\mu_{1},\mu_{2}\right)=\left(\frac{5}{2},\frac{10}{154}\right)$,
$F_{3}$: $\left(p_{1},p_{2}\right)=\left(\frac{99}{100},\frac{1}{100}\right)$ and $\left(\mu_{1},\mu_{2}\right)=\left(\frac{5}{2},\frac{10}{604}\right)$.

Each of them has a mean of 1. Therefore, the load remains the same, no matter which of the three is used. (The influence of the load was studied in the previous subsection, so now we prefer to keep it constant). $F_{1}-F_{3}$ have significantly different standard deviations, which are S=1.73, 4.28, and 8.50, respectively. In every case, X(0)=30, λ=1.1 and function $d_{4}$ are used.

**Figure 8:**
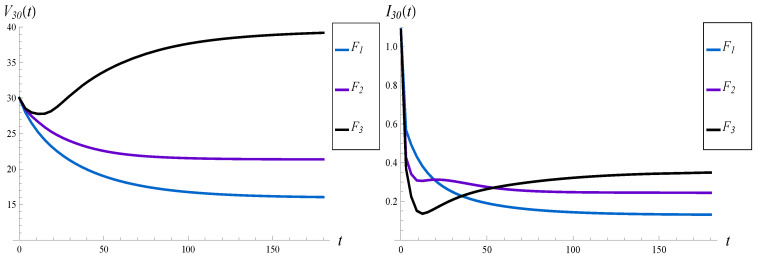
Average waiting time (on the left) and intensity of losses (on the right) vs. time, for different distributions of the service time.

**Figure 9:**
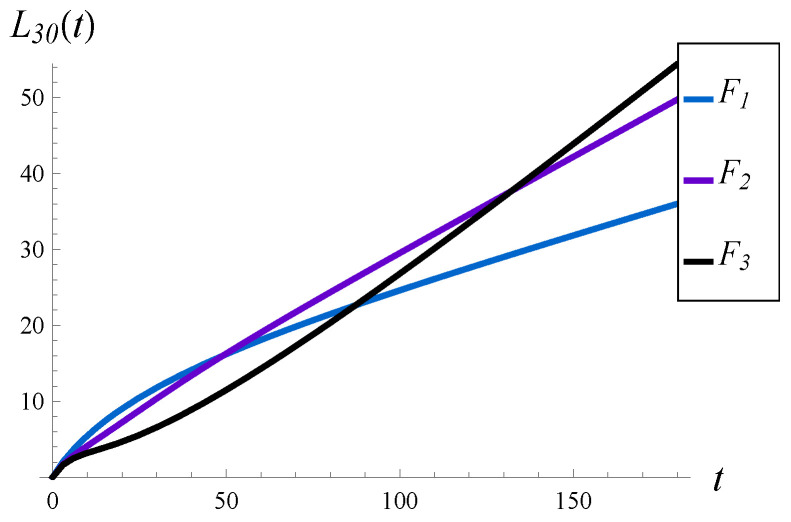
Average number of losses in (0,t) for different distributions of the service time.

The results for $F_{1}-F_{3}$ are depicted in Figure 8 and Figure 9. At least three observations can be made in these figures.

Firstly, the value of *S* has a profound impact on the transient characteristics of the system, especially the workload. Compare, for instance, black curves on the left-hand sides of Figure 6 and Figure 8. In Figure 8, the workload is greater in the whole interval t∈(0,∞) compared to Figure 6, even though the load is much higher in Figure 6 compared to Figure 8. Namely, we have ρ=1.5 versus ρ=1.1, and $F_{1}$ versus $F_{3}$ for the black curves, respectively, while other parameters are the same. In other words, enlarging the standard deviation has in this case a deeper impact on the transient workload than enlarging the queue load.

Secondly, the value of *S* seems to have a minor impact on the convergence time of the steady state. For all three distributions, $F_{1}$–$F_{3}$, this time seems to be similar.

**Figure 10:**
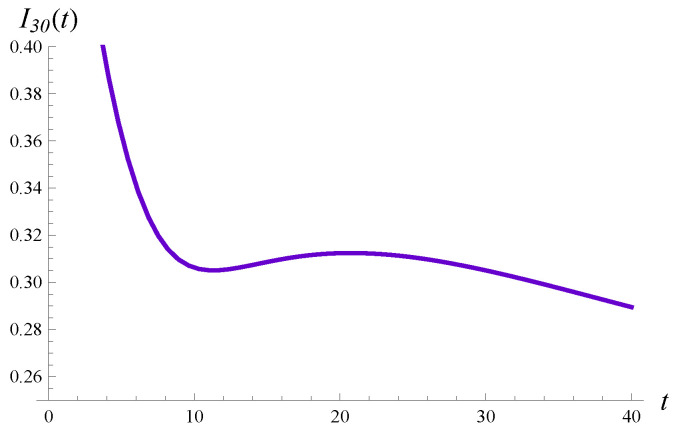
The intensity of losses vs. the time for the service time distribution $F_{2}$.

Thirdly, both the workload and the intensity of losses may or may not be monotonic in time, depending on *S*. As we can observe in Figure 8, one curve for the workload, and two curves for the intensity of losses, are not monotonic. In one case, we have two extrema. The extrema are visible more clearly in Figure 10, in which the transient intensity of losses for the distribution $F_{2}$ in the interval t∈(0,40) is depicted.

### 8.5. Verification via Simulations

Theoretical results proven in Section 5 and Section 6 were also verified using computer simulations. For this purpose, OMNeT++ was used [40]. OMNeT++ is a modular simulation framework, based on C++ language, designed mainly with network simulations in mind. It is available under the Academic Public License.

For the purpose of this research, the queuing system of Section 3 was implemented in OMNeT++ ver. 5.6, allowing the arbitrary configuration of the function assigning dropping probabilities to queue lengths, inter-arrival time distributions, service time distribution, and the buffer sizes.

Some special features of OMNeT++ were used due to the fact that transient simulations differ significantly from the traditional, steady-state simulations. In the latter case, the simulator works constantly for some (not short) time, enabling thousands of measurements of the characteristic of interest within one simulation run. In the transient case, the simulator works for a short time only, until a predefined *t* is reached. In the end, only one measurement of the characteristic of interest is taken and the simulation must be restarted, with the restored initial state of the system. Fortunately, all of these can be automatized in OMNeT++ using the *repeat* command in the configuration file. Furthermore, a lightweight user interface, called *cmdenv*, can be used in repeated simulations. It reduces to the absolute minimum the overhead related to the need of restarting the simulator over and over again.

With the help of these functionalities, $10^{5}$ simulation runs were performed to obtain every average waiting time and number of losses. The total execution time (all runs) depended on the assumed *t*, but was within the range of a few minutes on an average PC. All the simulations were performed using function $d_{1}$ for dropping packets, $F_{1}$ distribution of the service time, the system load of 1.1 and X(0)=30.

The results are presented in Table 2 and Table 3, for the transient waiting times and numbers of losses, respectively. As we can see, simulation results agree very well with their theoretical counterparts.

## 9. Conclusions

In this paper, the time-dependent analysis of the AQM scheme with rejections based on the queue length was carried out. A few important transient characteristics were derived—the average virtual waiting time, the average number of losses in a given interval and the intensity of losses. As side results, the stationary waiting time, the stationary loss probability, and the joint distribution of the number of accepted packets and losses in a given interval (assuming the service was suspended) were obtained.

Several numerical examples were presented, in which the evolution of the waiting times and losses were shown, depending on function *d*, initial buffer occupancy, queue load, and service time distribution. A few observations were made, some of them rather counterintuitive.

In particular, it was demonstrated that function *d* has a deep influence on the transient behavior of the waiting time and losses. Moreover, the more aggressive it is, the quicker is the convergence to the steady state. Both of these were to be expected. What was much more surprising was the fact that both the average waiting time and intensity of losses could be practically identical for two significantly different functions *d*, on the whole time axis t∈(0,∞).

It was shown also that the evolution in time of the average waiting time and intensity of losses may have sometimes a non-monotonic form, with multiple extrema.

Finally, it was demonstrated that the convergence time to the steady state is not a monotonic function of the queue load. Namely, a slower convergence was observed for the critical load of 1, rather than for a high or a low load.

## Figures and Tables

**Table 1:** Coefficients in the Zakian inversion method.

*j*	$w_{j}$	$u_{j}$
1	−36902.08210 + *i* 196990.4257	12.83767675 + *i* 1.666063445
2	61277.02524 − *i* 95408.62551	12.22613209 + *i* 5.012718792
3	−28916.56288 + *i* 18169.18531	10.93430308 + *i* 8.409673116
4	4655.361138 − *i* 1.901528642	8.776434715 + *i* 11.92185389
5	−118.7414011 − *i* 141.3036911	5.225453361 + *i* 15.72952905

**Table 2:** Theoretical vs. simulated average waiting times at *t*.

*t*	1	2	5	10	20	50	100	200	500
theoretical $V_{n}\left(t\right)$	29.05	28.20	26.03	23.36	20.04	15.91	14.17	13.79	13.75
simulated $V_{n}\left(t\right)$	29.02	28.18	26.01	23.34	20.04	15.95	14.12	13.83	13.75

**Table 3:** Theoretical vs. simulated average number of losses in (0,t).

*t*	1	2	5	10	20	50	100	200	500
theoretical $L_{n}\left(t\right)$	1.04	1.99	4.46	7.63	11.95	19.41	27.79	42.44	85.64
simulated $L_{n}\left(t\right)$	1.03	2.00	4.46	7.62	11.94	19.40	27.80	42.44	85.59

## Data Availability

Not applicable.
